# Systolic inter-arm blood pressure difference and estimated glomerular filtration rate in type 2 diabetic patients in Palestine: a cross-sectional study

**DOI:** 10.1080/07853890.2023.2259927

**Published:** 2023-09-25

**Authors:** Raghad Sweity, Khadeeja Fanoun, Tareq Jarrar, Bayan F. Alqtishat, Mohammad Abdelhafez, Suheir Ereqat

**Affiliations:** a Medical Research Club, Faculty of Medicine, Al-Quds University, Jerusalem, Palestine; b Department of Internal Medicine, Faculty of Medicine, Al-Quds University, Abu Dis, Jerusalem, Palestine; c Biochemistry and Molecular Biology Department, Faculty of Medicine, Al-Quds University, Abu Dis, Jerusalem, Palestine

**Keywords:** Type 2 diabetes mellitus, estimated glomerular filtration rate, chronic kidney disease, systolic inter-arm blood pressure difference, diabetes complications

## Abstract

**Objectives:**

This study aimed to investigate the association between systolic inter-arm blood pressure difference (IABPD) and the estimated glomerular filtration rate (eGFR), as well as chronic kidney disease (CKD), in patients with type 2 diabetes mellitus (T2DM).

**Patients and methods:**

This cross-sectional study included 189 Palestinians diagnosed with T2DM. Data were collected through personal interviews, medical records and three separate blood pressure measurements from both arms. Patients were stratified in two ways: based on systolic IABPD ≥15 mmHg and the presence of CKD, indicated by an eGFR of <60 mL/min/1.73 m^2^ over a three months period. We used simple and multiple linear regression analyses to clarify the association between systolic IABPD (mmHg) and eGFR and to identify independent predictors for eGFR.

**Results:**

The mean age was 61.3 years, with a female percentage of 57.7%. The prevalence of systolic IABPD ≥15 mmHg and CKD was 27.5% and 30.2%, respectively. Among patients with eGFR <60 mL/min/1.73 m^2^, the median systolic IABPD was 12.5 mmHg (interquartile range (IQR), 13.5 mmHg), whereas in patients with eGFR ≥60 mL/min/1.73 m^2^, it was 7.5 mmHg (IQR, 9.8 mmHg) with a significant difference (*p* = .021). The results of the multiple linear regression model did not reveal an independent association between systolic IABPD and eGFR, with an unstandardized coefficient (*B*) of −0.257 (95% confidence interval (CI), −0.623 to 0.109; *p* = .167). However, older age (*B*, −0.886; 95% CI, −1.281 to −0.49; *p* < .001), hypertension (*B*, −12.715; 95% CI, −22.553 to −2.878; *p* = .012) and a longer duration of DM (*B*, −0.642; 95% CI, −1.10 to −0.174; *p* = .007) were significantly and negatively associated with eGFR.

**Conclusions:**

Systolic IABPD did not exhibit an independent association with eGFR in T2DM patients. However, older age, a previous history of hypertension, and a longer duration of DM were all significantly associated with lower eGFR.

## 1. Introduction

Diabetes mellitus (DM) is one of the world’s most common rapidly growing endocrine diseases [1,2]. Its global prevalence was 10.5% in 2021 and may escalate to 12.2% in 2045 according to the International Diabetes Federation estimates [3]. While the Arab countries in the Middle East and North African regions are known for the highest prevalence, the Palestinian society rates are among the highest with a prevalence rate estimated at 15.3–20.8% in 2020 and could rise to 23.4% in 2030 [4,5]. DM is associated with many complications related to its effect on vessels, leading to an increase in micro- or macro-vascular disorder [6]. Within Palestinian society, DM-associated complications have been linked to around 5.7% of all deaths in Palestine, ranking as the 6th cause of death [7,8]. High on the list of infamous complications is DM-related chronic kidney disease (CKD), which besides hypertension was shown to be the two most common causes of CKD in the United States, all high- and middle-income countries, and many low-income countries [9,10]. CKD is defined as structural or functional abnormalities of the kidney. More specifically, it is defined as a decrease in kidney function shown by a glomerular filtration rate of less than 60 mL/min per 1.73 m^2^, or markers of kidney damage, or both, for at least 3 months, irrespective of the underlying etiology [11]. This definition applies to all causes of CKD. Patients with DM-related CKD are at increased risk of cardiovascular diseases and mortality [12,13]. DM-related CKD can be associated with many findings relating to the disease’s progression. Of interest, is the systolic inter-arm blood pressure difference (IABPD), defined as ≥10 mmHg difference in systolic blood pressure between both arms [14,15]. Therefore, if the difference is <10 mmHg, this is considered insignificant and within the normal range arising from anatomical variation between the right and left sides of the body [16]. According to the guidelines from National Institute of Health and Clinical Excellence (NICE), a 10-mmHg difference in systolic blood pressure between arms may be considered normal, while a difference of 15 mmHg may indicate an increased risk of vascular diseases [17]. Diabetic patients have a higher prevalence of systolic IABPD ≥10 mmHg than non-diabetic patients; as its prevalence was found to be 7.4% and 3.6% respectively in these groups [18]. However, patients with diabetes have other factors that may contribute to this finding such as high body mass index (BMI) and hypertension [19]. Systolic IABPD is more prevalent in patients with hypertension or previous cardiovascular diseases [20]. It is also associated with an increased risk of death and vascular problems predominantly peripheral arterial disease in patients with type 2 diabetes mellitus (T2DM) [19–21]. In patients with CKD, a systolic IABPD greater than 15 mmHg was found to be an independent factor in predicting the future of CKD in the general population [22]. It was also shown that systolic IABPD is high in patients with CKD who developed a cardiovascular event, with equal prevalence in all stages of CKD [23]. Patients with diabetes who developed diabetic complications like nephropathy have significant systolic IABPD [14]. Retinopathy is another diabetic complication associated with high systolic IABPD, which is associated with subclinical atherosclerosis that could be a contributing cause of retinopathy in this population [10]. Diabetic patients with systolic IABPD have increased atherosclerotic markers, which could explain the association between systolic IABPD and the diabetic complications [14]. IABPD reflects the macrovascular complications of T2DM including hypertension, which could contribute to the development of microvascular complications such as nephropathy; this is explained by the development of glomerular hyperfiltration because of the impaired autoregulation of the glomerular afferent arteriole as a result of the vascular events [24]. The effect of systolic IABPD on the development of organ damage remains controversial with no studies focused on the effect of systolic IABPD on the development of CKD in T2DM patients. This study aims to examine the prevalence and factors associated with both abnormal systolic IABPD and an estimated glomerular filtration rate (eGFR) of less than 60 mL/min per 1.73 m^2^, and to investigate the association between systolic IABPD and eGFR, which in turn reflects the development of CKD.

## 2. Methods

### 2.1. Study design and population

This cross-sectional study was conducted between August 2021 and June 2022 at the Ministry of Health primary healthcare centres and governmental hospitals in Hebron, Ramallah and Bethlehem districts. The study included 189 hospitalized and outpatient adults above 18 years old with an established diagnosis of T2DM and stable retrospective serum creatinine (SCr) for more than three months. We excluded patients with diabetes types other than type 2, as well as those with malignancies that may interfere with diabetes progression (pancreas, stomach or kidney). Additionally, patients who had been diagnosed with upper limb vasculature abnormalities, subclavian artery stenosis or aortic dissection were also excluded. All patients involved in this study were invited to participate on a voluntary basis after the aim of the research and the procedures for data collection and blood pressure measurement were clarified. All participants gave informed consent and the study procedure was approved by the Ministry of Health and the Research Ethics Committee at Al-Quds University (Ref#:162/1059/2021).

### 2.2. Data collection and blood pressure measurements

Patients’ data were collected from their medical records and laboratory tests. A questionnaire was used to collect data from both personal interviews and the medical records. The collected data included demographics, smoking status, BMI, medical history, biochemical tests (SCr, glycated haemoglobin, blood urea nitrogen and lipid profile) and medication history. Blood pressure measurements were systematically obtained from both arms after at least 5 min of rest in the sitting position. Three bilateral measurements were taken in succession, beginning with the right arm and then the left one. Between each consecutive measurement of both arms, there was a 2-min resting period. The patient’s arm was kept at the level of the heart during the measurement. Blood pressure readings were obtained by qualified personnel using a standardized electronic sphygmomanometer, the ‘*Dinamap Mindray VS-600*’. We calculated the average of the three readings of the systolic and diastolic BPs in both arms. The systolic IABPD was identified as the difference between the average systolic blood pressure in the right and left arms. Based on NICE guidelines, the systolic IABPD ≥15 was considered abnormal. Quantitative measurement of urine albumin was not available because it is not routinely done in Palestinian Ministry of Health hospitals and clinics; therefore, we only functionally calculated the eGFR using the CKD Epidemiology Collaboration formula (CKD-EPI) based on SCr, which is the most accepted and widely used equation that has been shown to have a better estimation ability of the eGFR compared to other formulas [25,26]. Based on the calculated eGFR, and according to the international guideline group Kidney Disease Improving Global Outcomes, we defined CKD as eGFR of less than 60 mL/min/1.73 m^2^ for more than three months [27].

### 2.3. Statistical analysis

Patient characteristics were summarized using descriptive statistics. Continuous variables were expressed as mean and standard deviation (SD), while categorical variables were expressed as frequencies and percentages. To identify factors associated with systolic IABPD ≥15 mmHg and factors associated with a baseline eGFR <60 mL/min/1.73 m^2^, we stratified the patients according to their systolic IABPD and eGFR. The *χ*^2^-test was used to compare categorical variables. Continuous variables for the subgroups were expressed as the median and interquartile range (IQR); additionally, we used the Shapiro–Wilk test to assess the normality of the continuous variables. Accordingly, the independent sample *t*-test or Wilcoxon’s rank-sum test was used to compare continuous variables. To identify the association between systolic IABPD and eGFR, we initially used simple linear regression to identify factors associated with eGFR. Subsequently, we performed multiple linear regression, adjusting for all variables with a significance level of *p* < .05 as determined in the simple linear regression. Additionally, we incorporated adjustments for BMI and dyslipidaemia into the model, as these factors are recognized risk factors for CKD [28]. To identify potential multicollinearity among the independent variables in the regression model, we utilized the variance inflation factor (VIF). We conducted a post hoc analysis utilizing G*Power 3.1 to evaluate the statistical power of our findings. Comprehensive details are provided in Supplemental Table A. Multiple imputations were used to replace missing values (Supplemental Table B) when the percentage of missing values was at least 5%, assuming that they were missing at random [29]. Twenty copies of the dataset were generated with the missing values replaced by imputed values based on participant-related characteristics. Non-normally distributed variables were first transformed using log transformation, and the data were transformed back to the original scale after imputations. Variables used in the imputation phase include age, sex, BMI, duration of DM, smoking, history of dyslipidaemia, the use of a lipid-lowering agent, HbA1C, cholesterol, TG, LDL, HDL and FBS. Using Rubin’s rule, the results from each dataset were combined to produce a final result, which was applied to the final pooled dataset to perform a bivariate analysis. SPSS version 26.0 (SPSS Inc., Chicago, IL) and the R environment version 4.1.3 (Vienna, Austria) were used for data analysis. Statistical significance was set at *p* < .05. Multiple imputation analyses were performed using the ‘mice’ package.

## 3. Results

### 3.1. Demographic and clinical characteristics of study participants

This study included 189 T2DM patients with a mean age of 61.3 (SD, 11.3) and a mean DM duration of 11.6 (SD, 9.1) years. Of them, 109 (57.7%) were female patients; 43 (22.8%) were smokers; 124 (64%) had a history of dyslipidaemia; 129 (68.3%) have hypertension; 86 (45.5%) have cardiovascular diseases (including coronary artery diseases and heart failure); and 68 (36%) have diabetic retinopathy. The prevalence of CKD based on eGFR was 30.2% (95% confidence interval (CI), 22.7–37.5%), and the prevalence of systolic IABPD ≥15 mmHg was 27.5% (95% CI, 20.8–34.2%). Table 1 shows the patients’ demographics, their clinical characteristics and the medication classes they are currently using.

**Table 1.** Baseline demographic and clinical characteristics.

Variable	
Age (years)	61.3 ± 11.3
Sex, females (*n*, %)	109, 57.7
Duration of DM (years)	11.6 ± 9.1
Current smoker (*n*, %)	43, 22.8
History of dyslipidaemia (*n*, %)	124, 64
BMI (kg/m^2^)	31.1 ± 6.9
HbA1C (%)	8.3 ± 2.2
FBS (mg/dL)	201.1 ± 93.7
Creatinine (mg/dL)	1.5 ± 1.4
eGFR	71.9 ± 32.1
Diabetic retinopathy (*n*, %)	68, 36
Hypertension disease	129, 68.3
Systolic blood pressure (mmHg)	139.2 ± 24.9
Diastolic blood pressure (mmHg)	76.6 ± 13.2
Cholesterol (mg/dL)	165.8 ± 44.5
TG (mg/dL)	175.2 ± 117.7
LDL (mg/dL)	97.3 ± 57.8
HDL (mg/dL)	46.9 ± 40.7
BUN (mg/dL)	33.8 ± 23.0
Medications (*n*, %)	
Metformin	134, 70.9
Glimepiride	36, 19
Insulin	99, 52.4
Lipid-lowering medications	130, 68.8
Antihypertensive medications	118, 62.4
Angiotensin-converting enzyme inhibitors	19, 10.1
Angiotensin receptor blockers	16, 8.5
Beta blockers	48, 25.4
Alpha blockers	3, 1.6
Calcium channel blockers	38, 20.1
Unspecified antihypertensive drug^a^	34, 18
Diuretics	18, 9.5
Aspirin	87, 46

DM: diabetes mellitus; BMI: body mass index; HbA1c: glycated haemoglobin; FBS: fasting blood sugar; BP: blood pressure; IABPD: inter-arm blood pressure difference; eGFR: estimated glomerular filtration rate; TG: triglyceride; HDL: high density lipoprotein; LDL: low density lipoprotein; BUN: blood urea nitrogen.

^a^ Patients with unspecified antihypertensive drugs in their records and cannot recall the name or type of their antihypertensive medication. Continuous variables are expressed as mean ± standard deviation.

### 3.2. Systolic inter-arm blood pressure difference

The study participants were stratified into two groups according to the systolic IABPD: 52 (27.5%) patients with systolic IABPD ≥15 mmHg and 137 (72.5%) patients with systolic IABPD <15 mmHg. As presented in Table 2, the median values for systolic blood pressure, diastolic blood pressure and eGFR displayed significant differences between the two groups (*p* < .05). The median eGFR for patients with systolic IABPD ≥15 mmHg was 65 (IQR, 49) mL/min/1.73 m^2^, whereas for patients with systolic IABPD <15 mmHg, it was 84 (IQR, 42) mL/min/1.73 m^2^ (*p* = .013). However, no significant differences were observed in the duration of diabetes, history of cardiovascular disease, dyslipidaemia, hypertension, as well as in BMI, HbA1c, FBS, TG, cholesterol, HDL or LDL between the two groups.

**Table 2.** Patients’ characteristics stratified according to systolic inter-arm blood pressure difference.

	Systolic IABPD ≥ 15 mmHg (no. = 52)	Systolic IABPD < 15 mmHg (no. = 137)	*p* Value
Age (years)	63.5 (16.5)	60 (14)	.117^b^
Sex, female (*n*, %)	30, 57.7	79, 57.7	1
Duration of DM (years)	13 (15)	9 (11.3)	.09
Smoking (*n*, %)	11, 21.2	32, 23.4	.747
BMI (kg/m^2^)	29.7 (8.6)	30.5 (9)	.594
Hypertension (*n*, %)	39, 75	90, 65.7	.22
Systolic BP (mmHg)	144.8 (40.8)	131.7 (48.8)	**<.001**
Diastolic BP (mmHg)	79.5 (20.1)	73 (15)	**.006**
CKD^a^ (*n*, %)	22, 42.3	35, 25.5	**.025**
Dyslipidaemia (*n*, %)	35, 67.3	94, 68.6	.863
History of cardiovascular disease (*n*, %)	28, 53.8	58, 42.3	.156
Laboratory tests			
HbA1C (%)	7.9 (2.1)	8 (3)	.852
FBS (mg/dL)	198.5 (160.9)	167 (114.4)	.118
eGFR (mL/min/1.73 m^2^)	65 (49)	84 (42)	**.013**
TG (mg/dL)	143 (73.4)	150 (103)	.470
Cholesterol (mg/dL)	157 (62.7)	164.7 (49.9)	.472
HDL (mg/dL)	43.4 (18.7)	40 (17.7)	.417
LDL (mg/dL)	93.6 (47)	86.7 (46.3)	.765
BUN (mg/dL)	27.4 (34.7)	26.6 (19.1)	.420

IABPD: inter-arm blood pressure difference; DM: diabetes mellitus; BMI: body mass index; HbA1c: glycated haemoglobin; FBS: fasting blood sugar; BP: blood pressure; CKD: chronic kidney disease; eGFR: estimated glomerular filtration rate; TG: triglyceride; HDL: high-density lipoprotein; LDL: low-density lipoprotein; BUN: blood urea nitrogen.

*p* values are highlighted in bold if they fall below the significance threshold of .05.

^a^ Chronic kidney disease (CKD) was defined by a sustained estimated glomerular filtration rate (eGFR) of less than 60 mL/min/1.73 m^2^ over a minimum duration of three months. Continuous variables are expressed as the median (interquartile range), and a *p* value was obtained using the Wilcoxon rank-sum test.

^b^ *p* Value was obtained by the independent sample *t*-test. For the categorical variables, the *p* value was obtained by *χ*^2^-test.

### 3.3. Estimated glomerular filtration rate

We stratified the study participants into two groups based on eGFR: 57 patients with eGFR < 60 mL/min/1.73 m^2^ (CKD patients) and 132 patients with eGFR ≥ 60 mL/min/1.73 m^2^ (non-CKD patients). Our results showed that age, sex, duration of DM, hypertension, systolic blood pressure, systolic IABPD and history of cardiovascular disease were significantly different between the two groups (*p* < .05) (Table 3). Although the prevalence of diabetic retinopathy did not show a significant difference between the two groups, the median eGFR for patients with diabetic retinopathy was 69 (IQR, 42.8), which was significantly lower compared to patients without diabetic retinopathy at 83 (IQR, 52), *p* = .026. The results of the simple linear regression with eGFR as the dependent variable are provided in Supplemental Table C. In the multiple linear regression model, systolic IABPD, systolic blood pressure, age, sex, history of hypertension, duration of DM, smoking, history of cardiovascular disease, and history of diabetic retinopathy were included as independent variables. The model was further adjusted for BMI and dyslipidaemia. The model’s coefficient of determination (*R*^2^) and adjusted coefficient of determination (adjusted *R*^2^) were found to be 0.368 and 0.327, respectively. Our results did not reveal an independent association between systolic IABPD (mmHg) and eGFR, as indicated by the unstandardized coefficient (*B*) of −0.257 (95% CI, −0.623 to 0.109; *p* = .167). However, older age (*B*, −0.886; 95% CI, −1.281 to −0.490; *p* < .001), hypertension (*B*, −12.715; 95% CI, −22.553 to −2.878; *p* = .012) and a longer duration of DM (*B*, −0.642; 95% CI, −1.110 to −0.174; *p* = .007) were found to be significantly and negatively associated with eGFR. There was no evidence of multicollinearity between the independent variables (VIF <5). The details of the fitted model, including unstandardized coefficients (*B*) and standardized coefficients (Beta), are presented in Table 4.

**Table 3.** Patients’ characteristics stratified according to chronic kidney disease.

	CKD, eGFR < 60 mL/min/1.73 m^2^ (no. = 57)	Non-CKD, eGFR ≥ 60 mL/min/1.73 m^2^ (no. = 132)	*p* Value
Age (years)	68 (13)	59 (13)	**<.001** ^a^
Sex, female (*n*, %)	39, 68.4	70, 53.0	**.049**
Duration of DM (years)	15 (11)	7 (11.3)	**<.001**
Smoking (*n*, %)	11, 19.3	32, 24.2	.457
BMI (kg/m^2^)	30.9 (8.5)	29.7 (8.7)	.9
Hypertension (*n*, %)	51, 89.5	78, 59.1	**<.001**
Systolic BP (mmHg)	145 (38.7)	132.17 (29.8)	**.004**
Diastolic BP (mmHg)	71.33 (22)	75.7 (14.2)	.1
Systolic IABPD ≥15 mmHg	22, 38.6	30, 22.7	**.025**
Systolic IABPD (mmHg)	12.5 (13.5)	7.5 (9.8)	**.021**
Dyslipidaemia (*n*, %)	38, 66.7	86, 65.2	.869
History of cardiovascular disease (*n*, %)	38, 66.7	48, 36.4	**<.001**
Diabetic retinopathy (*n*, %)	45, 34.1	23, 40.4	.511
Laboratory tests			
HbA1C (%)	7.6 (2.3)	8.2 (2.8)	.072
FBS (mg/dL)	176.7 (90.3)	168.9 (144.8)	.948
TG (mg/dL)	143 (99.6)	150.5 (102.6)	.979
Cholesterol (mg/dL)	160.7 (71.7)	161.5 (46.3)	.880
HDL (mg/dL)	39.3 (16.5)	41.7 (18.8)	.736
LDL (mg/dL)	93.6 (62.9)	88.1 (36.6)	.980
BUN (mg/dL)	48.7 (48.4)	23.8 (15.9)	**<.001**

CKD: chronic kidney disease; DM: diabetes mellitus; BMI: body mass index; HbA1c: glycated haemoglobin; FBS: fasting blood sugar; BP: blood pressure; IABPD: inter-arm blood pressure difference; eGFR: estimated glomerular filtration rate; TG: triglyceride; HDL: high-density lipoprotein; LDL: low-density lipoprotein; BUN: blood urea nitrogen.

Continuous variables are expressed as the median (interquartile range), and a *p* value was obtained using the Wilcoxon rank-sum test.

*p* values are highlighted in bold if they fall below the significance threshold of .05.

^a^ *p* Value was obtained by the independent sample *t*-test. For the categorical variables, the *p* value was obtained by *χ*^2^-test.

**Table 4.** Multiple linear regression model to identify independent predictors of estimated glomerular filtration rate.

Variables^a^	Unstandardized coefficients^b^		Standardized coefficients^c^			
	*B*	95% confidence interval	Beta	*t*	*p* Value	*VIF*
Systolic IABPD (mmHg)	−0.257	−0.623 to 0.109	−0.094	−1.388	.167	1.24
Systolic BP (mmHg)	−0.108	−0.293 to 0.077	−0.086	−1.153	.251	1.48
Hypertension (yes)	−12.715	−22.553 to −2.878	−0.399	−2.551	**.012**	1.44
Age (years)	−0.886	−1.281 to −0.490	−0.304	−4.422	**<.001**	1.27
Sex (female)	−4.533	−13.731 to 4.665	−0.142	−0.973	.332	1.42
Duration of DM (years)	−0.642	−1.110 to −0.174	−0.186	−2.707	**.007**	1.27
BMI (kg/m^2^)	0.122	−0.469 to 0.713	0.027	0.408	.684	1.15
Smoking (yes)	4.884	−6.401 to 16.168	0.153	0.854	.394	1.54
History of CVD (yes)	−4.289	−12.558 to 3.981	−0.135	−1.024	.307	1.16
Diabetic retinopathy (yes)	−0.43	−8.902 to 8.042	−0.014	−0.1	.92	1.12
Dyslipidaemia (yes)	−1.09	−9.871 to 7.692	−0.034	−0.245	.807	1.14
*R*^2^ = 0.368, Adj. *R*^2^ = 0.327						

VIF: variance inflation factor; IABPD: inter-arm blood pressure difference; BP: blood pressure; DM: diabetes mellitus; CVD: cardiovascular disease.

*p* Values are highlighted in bold if they fall below the significance threshold of .05.

^a^ Categorical variables were entered into analysis using dummy coding.

^b^ Unstandardized coefficient (*B*) signifies the extent of alteration in a dependent variable (eGFR) resulting from a 1-unit shift in each respective independent variable.

^c^ Standardized coefficient (Beta) measures the relative influence of individual independent variables on the dependent variable. A higher absolute value of the beta coefficient indicates a stronger effect.

## 4. Discussion

High systolic blood pressure is an important risk factor for CVD, peripheral arterial disease and diabetes-related mortality. The prevalence of systolic IABPD and its effect on the occurrence of organ damage has been investigated in several populations. In this context, our study aimed to explore the prevalence of systolic IABPD ≥15 mmHg among type 2 diabetic patients in Palestine and examine its association with eGFR and the development of CKD.

Our results revealed that the prevalence of systolic IABPD ≥15 mmHg was 27.5%. The high prevalence of systolic IABPD ≥15 mmHg indicates that these patients could be at risk of vascular events and hence in need of regular follow-up.

The definition of significant systolic IABPD varied according to previous reports and the measuring methods, with systolic IABPD ≥10 mmHg being the traditional cut-off value. Although we defined a significant systolic IABPD in our study at 15 mmHg or higher, according to the NICE recommendations [17], the prevalence of significant systolic IABPD was higher in our study compared to previous reports that used a cutoff of 10 mmHg. A cross-sectional study was conducted to investigate the association between DM and systolic IABPD, it was found that the prevalence of systolic IABPD ≥10 mmHg in the diabetic population was 8.4% compared to 5.4% in nondiabetics, but the difference was not significant after the adjustment for other confounders [19]. These findings were similar to the findings of Clark et al. study, which found that the prevalence of systolic IABPD ≥10 mmHg in patients with diabetes was 8.6%, which was associated with peripheral artery disease, but a systolic IABPD ≥15 mmHg was associated with diabetic retinopathy and nephropathy [30]. Another study, involving 800 patients with T2DM, used cut-off values of 5, 10 and 15 mmHg and separately examined systolic and diastolic IABPD. Their findings indicated prevalence of 43.8%, 13.4% and 4.6%, respectively [31].

This variation may be attributed to the significant difference in the sample size, which was smaller at 189 patients in our study compared to 700 or more in these studies; it also may be attributed to the sequential approach of blood pressure measurement that we have used compared to the simultaneous approach adopted by these studies [19,30,31]. The patient’s position and the type of device used in measuring blood pressure can also affect the level of systolic IABPD. Interestingly enough, studies conducted on the healthy general population found a high prevalence of systolic IAPBD ≥10 or ≥15. These studies include a cross-sectional study by Seethalakshmi and Bahuleyan, which examined 110 healthy Indian individuals and reported a prevalence of 46% for Systolic IABPD >10 among young, healthy individuals. It also concluded that this condition is associated with a higher risk of CVD [32]. Another cross-sectional study, conducted by Essa et al. in the Kurdistan Region of Iraq, found a prevalence of 37.1% for the same systolic IABPD range among 3030 young and healthy volunteers included in the study [33]. Systolic IABPD ≥15 was also found to be highly prevalent (approximately 17%) in another study that enrolled 1634 healthy individuals [34].

The prevalence of CKD among T2DM patients in our study was 30.2%, which is similar to what was reported in a meta-analysis conducted on nine studies that showed that the pooled estimated prevalence of CKD in patients with DM in the Middle East was 28.96% (95% CI, 19.80–38.11) [35]. Despite this, the unavailability of the albumin-to-creatinine ratio and the sole reliance on eGFR may lead to an underestimation of the true prevalence of CKD among our study sample.

The association between systolic IABPD and CKD has been the subject of numerous studies. A retrospective cohort study involving 8780 was conducted on adults from the general Korean population and showed that increased systolic IABPD is an independent predictor to the development of CKD [22]. Another Korean retrospective study, which included 563 patients with type 2 diabetes, found that diabetic patients with systolic IABPD ≥5 and ≥10 mmHg showed a significant association with proteinuria, which is a marker of kidney damage [10]. On the other hand, studies that investigate the association between systolic IABPD and eGFR are scarce. A southern Taiwanese study that included 144 patients with stage 3–5 CKD found that an increased interankle systolic blood pressure difference was associated with a worse eGFR slope and rapid renal function decline [36]. Another study was conducted on the Chinese population to examine the association of four-limb blood pressure differences with target organ changes in the elderly. The study found that four-limb systolic blood pressure differences, including systolic IABPD, were independently associated with eGFR, indicating a potential link between blood pressure differences and kidney function [37]. In our study, we observed that median eGFR was lower among diabetic patients with systolic IABPD ≥15 mmHg than those with systolic IABPD <15 mmHg. However, multiple linear regression analysis revealed that systolic IABPD did not predict eGFR decline. These results could be explained by the presence of other factors found to predict eGFR decline, including older age, hypertension history, and a longer duration of DM. Among older ages, significant structural changes can occur in the kidneys’ vasculature, which in turn affect the filtration function, leading to a decline in eGFR [38]. Patients with a history of hypertension have a greater risk of diminishing kidney function. In a study involving 14,854 participants, Yu et al. found that patients with hypertension had faster eGFR declines than those without hypertension [39]. Regarding the duration of diabetes, a study examined the rate at which eGFR declines in individuals with T2DM. It found that patients with T2DM diagnosed at a younger age or those with a longer history of the disease might experience a more rapid decline in their eGFR compared to those diagnosed at middle age or with a shorter duration of diabetes [40].

To the best of our knowledge, this study is the first to investigate the prevalence of abnormal IABPD among T2DM Palestinian patients. The study participants were recruited from several medical centres and hospitals in Palestine, which may represent the diabetic Palestinian population. We also measured the blood pressure in both arms three times to ensure the accuracy of the measurement and decrease the incidence of false positives. Furthermore, we used a cut-off of 15 mmHg rather than 10 mmHg in concordance with the NICE guidelines.

However, our study had some limitations that should be considered. First, due to the relatively small sample size, the stratification of data based on CKD stages was not applicable for subgroup analysis. Another limitation was related to the sampling procedure. As the study participants were recruited from in- and out-patient clinics, the inpatient participants were older with multiple comorbidities, which may affect our findings on systolic IABPD and thus limit the generalizability of our results. The baseline comorbidities and past medical history of some patients were not available in their medical records and were obtained based on self-reported events, increasing the possibility of recall bias. The fact that we used the consecutive approach for the measurement of blood pressure may overestimate our findings on systolic IABPD. The unavailability of albumin-to-creatinine ratio data in patients’ records in our healthcare facilities, where this test is rarely conducted, may potentially result in an underestimation of the actual prevalence of CKD within our study sample. Several studies revealed that the white coat effect may also cause higher systolic IABPD in the sequential measurement and is associated with overestimation of the difference compared to the repeated simultaneous measurement [41,42]. Therefore, we recommend conducting a larger prospective cohort study that overcomes all these limitations to further determine the prevalence of systolic IABPD and to investigate the value of using the systolic IABPD as a predictor marker for the decline in eGFR in patients with T2DM.

## 5. Conclusions

The prevalence of the systolic IABPD at a cutoff value of ≥15 mmHg was high among Palestinians with T2DM. In addition, CKD patients, as defined by eGFR, exhibited significantly higher systolic IABPD. In the multiple linear regression analysis with eGFR as the dependent variable, systolic IABPD did not show an independent association with eGFR in T2DM patients, while older age, a previous history of hypertension, and a longer duration of DM were all significantly and inversely associated with eGFR.

## Supplementary Material

Supplemental Material

## Data Availability

Data set is available with the corresponding author and can be provided upon request.
