# Supplementary material for: Systolic inter-arm blood pressure difference and estimated glomerular filtration rate in type 2 diabetic patients in Palestine: a cross-sectional study
Source: Ann Med. 2023 Sep 25;55(2):2259927. doi: 10.1080/07853890.2023.2259927 (PMC10521340; doi:10.1080/07853890.2023.2259927)
Supplement: Supplemental Material [file IANN_A_2259927_SM8843.docx]

**Supplementary**

**Contents:**

Supplemental Table A: Posteriori power analysis

Supplemental Table B: Missing data per each variable

Supplemental Table C. Simple linear regression analysis of variables associated with estimated glomerular filtration rate

**Supplemental Table A: Posteriori power analysis**

| F tests - Linear multiple regression: Fixed model, R² deviation from zero | |
| --- | --- |
| Input: |  |
| Effect size f² | 0.4858841 |
| α err prob | 0.05 |
| Total sample size | 189 |
| Number of predictors | 11 |
| Output: |  |
| Noncentrality parameter λ | 91.8320949 |
| Critical F | 1.8430804 |
| Numerator df | 11 |
| Denominator df | 177 |
| Power (1-β err prob) | 1 |

Output from G*power 3.1 [computer program]

**Supplemental Table B: Missing data per each variable**

| Variable | Missing data n, % |
| --- | --- |
| Age, yr | 0, 0 |
| Sex, females n, % | 0, 0 |
| Duration of DM, yr | 1, 0.5 |
| Current smoker n, % | 0, 0 |
| History of dyslipidemia n, % | 7, 3.7 |
| BMI kg/m^2^ | 6, 3.2 |
| HbA1C % | 21, 11.1 |
| FBS mg/dl | 32, 16.9 |
| Creatinine mg/dl | 0, 0 |
| eGFR | 0, 0 |
| Systolic blood pressure mmHg | 0, 0 |
| Diastolic blood pressure mmHg | 0, 0 |
| Cholesterol mg/dl | 31, 16.4 |
| TG mg/dl | 31, 16.4 |
| LDL mg/dl | 42, 22.2 |
| HDL mg/dl | 36, 19.1 |
| BUN mg/dl | 3, 1.6 |
| Medications n, % |  |
| Metformin | 0, 0 |
| Glimepiride | 0, 0 |
| Insulin | 0, 0 |
| Lipid lowering drugs | 0, 0 |
| Antihypertensive drugs | 0, 0 |

yr, years; DM, diabetes mellitus; BMI, body mass index; HbA1c, Glycated hemoglobin; FBS, fasting blood sugar; BP, blood pressure; IABPD, inter-arm blood pressure difference; eGFR, estimated glomerular filtration rate; TG, triglycerides; HDL, high density lipoprotein; LDL, low density lipoprotein; BUN, blood urea nitrogen.

**Supplemental Table C: Simple linear regression analysis of variables associated with estimated glomerular filtration rate**

| Variables ^a^ | Unstandardized Coefficients ^b^ | | Standardized Coefficients ^c^ |  |  |
| --- | --- | --- | --- | --- | --- |
|  | B | 95% confidence interval | Beta | t | *P* value |
| Age, yr | -1.157 | -1.533 to -0.782 | -0.407 | -6.089 | **<0.001** |
| Sex, female | -10.193 | -19.422 to -0.964 | -0.318 | -2.179 | **0.031** |
| Duration of DM, yr | -1.274 | -1.749 to -0.799 | -0.362 | -5.29 | **<0.001** |
| Smoking, yes | 11.067 | 0.169 to 21.964 | 0.345 | 2.003 | **0.047** |
| BMI, kg/m^2^ | -0.268 | -0.941 to 0.404 | -0.058 | -0.787 | 0.432 |
| Hypertension, yes | -24.472 | -33.742 to -15.203 | -0.399 | -5.208 | **<0.001** |
| Systolic BP, mmHg | -0.312 | -0.493 to -0.132 | -0.243 | -3.423 | **0.001** |
| Systolic IABPD, mmHg | -0.566 | -0.961 to -0.171 | -0.203 | -2.83 | **0.005** |
| Diastolic BP, mmHg | 0.11 | -0.241 to 0.460 | 0.045 | 0.618 | 0.538 |
| History of CVD, yes | -16.916 | -25.861 to -7.970 | -0.527 | -3.73 | **<0.001** |
| Dyslipidemia, yes | -8.82 | -18.658 to 1.017 | -0.275 | -1.769 | 0.079 |
| Diabetic retinopathy, yes | -9.752 | -19.270 to -0.235 | -0.304 | -2.021 | **0.045** |
| HbA1c, % | 0.401 | -0.337 to 1.139 | 0.078 | 1.072 | 0.285 |
| FBS, mg/dl | 0.022 | -0.027 to 0.072 | 0.065 | 0.893 | 0.373 |
| Cholesterol, mg/dl | -0.047 | -0.151 to 0.057 | -0.066 | -0.898 | 0.371 |
| TG, mg/dl | -0.015 | -0.054 to 0.025 | -0.054 | -0.733 | 0.465 |
| HDL, mg/dl | 0.022 | -0.092 to 0.136 | 0.028 | 0.384 | 0.702 |
| LDL, mg/dl | -0.075 | -0.154 to 0.005 | -0.134 | -1.852 | 0.066 |

^a^ Categorical variables were entered into analysis using dummy coding.
 ^b^ Unstandardized coefficient (B) signifies the extent of alteration in a dependent variable (eGFR) resulting from a 1-unit shift in each respective independent variable.
 ^c^ Standardized coefficient (Beta) measures the relative influence of individual independent variables on the dependent variable. A higher absolute value of the beta coefficient indicates a stronger effect.
 *P* values are highlighted in bold if they fall below the significance threshold of 0.05. BP, blood pressure; yr, years; DM, diabetes mellitus; IABPD, inter-arm blood pressure difference; CVD, cardiovascular disease; HbA1c, Hemoglobin A1c; FBS, Fasting Blood Sugar; TG, Triglycerides; HDL, High-Density Lipoprotein; LDL, Low-Density Lipoprotein
